# Review of *Storytelling in Queer Appalachia: Imagining the Unspeakable Other*

**DOI:** 10.13023/jah.0403.07

**Published:** 2023-01-01

**Authors:** Sandra L. Cotton, Laurie A. Theeke, James Messer

**Affiliations:** West Virginia University, scotton@hsc.wvu.edu; George Washington University; West Virginia University

**Keywords:** Appalachia, book review, gender and sexuality studies

## Abstract

The Journal of Appalachian Health is committed to reviewing published media that relates to contemporary concepts affecting the health of residents of Appalachia. Improving the health in the region of Appalachia means knowing our people as they live and thrive in communities. The book reviewed here, *Storytelling in Queer Appalachia: Imagining the Unspeakable Other* (Edited by Glasby, Gradin, and Ryerson), is a must read for people who wish to gain insight on the real experience of being queer in Appalachia.

## MEDIA TYPE: BOOK

### CITATION

Glasby H, Gradin S, Ryerson R, editors. *Storytelling in queer Appalachia: imagining the unspeakable other*. First edition. Morgantown WV: West Virginia University Press, 2020. ISBN-13: 978-1949199482; Cost: $29.99 paperback, $25.49 e-Textbook Edition, $99.99 Hardcover.

### ABOUT THE REVIEWERS

**Dr. Sandra Cotton**, DNP, APRN-BC, FNAP, is Clinical Associate Professor at West Virginia University (WVU) School of Nursing’s Morgantown Campus, an LGBTQ+ community member, and Founding Chair of the WVU School of Nursing’s Diversity, Equity, and Inclusion (DEI) Committee. Dr. Cotton has over 40 years as an RN, 25 years as an APRN, and previously worked for the AIDS Clinical Trials Unit at Johns Hopkins University at the height of the 1980s AIDs crisis. She has been a nurse educator for nearly 30 years at an Appalachian land-grant university.

**Dr. Laurie Theeke**, PhD, RN, FNP-BC, GCNS-BC, FNAP, FAAN, currently serves as the Associate Dean for the PhD in Nursing program at the George Washington University School of Nursing. Dr. Theeke is a native Appalachian, LGBTQ+ mother, sister, and ally, and a founding member of the WVU School of Nursing’s DEI Committee. Dr. Theeke has over 30 years of experience as a nurse practitioner caring for people from West Virginia. She has served as a nurse educator since 2008, teaching graduate nurses in clinical graduate programs and PhD courses. Dr. Theeke is internationally known for her expertise on loneliness, social isolation, and its impact on psychological and physical health outcomes.

**James Messer**, MSN, RN, CNE, is Assistant Professor of Clinical Education at WVU School of Nursing, Beckley Campus. A native Appalachian, he is an LGBTQ+ ally and founding member of the WVU School of Nursing’s DEI Committee. James is a faith-community and mental health nurse. He strives to promote true equality through his work.

### ABOUT THE AUTHORS

Hillery Glasby is a professor of writing at Michigan State University. Dr. Glasby’s program of research focuses on LGBTQ+ student writers and queer rhetoric. Both Sherrie Gradin and Rachael Ryerson are affiliated with Ohio University. Dr. Gradin taught courses on queer studies and writing, with a focus on rhetoric and composition. Dr. Ryerson has known expertise on gender studies. This book presents a unique compilation of stories, exploring how sexual identity is lived in rural Appalachia.

### THE REVIEW

This book presents a collection of essays selected with the goal of employing stories to amplify the voices of queer Appalachians—to make queerness visible. It is an essential work for people who wish to gain insight on the real experience of being queer in Appalachia. The authors, who are from diverse walks of life—being practicing activists, advocates, scholars, students, and divinity writers—explicate the complexity of Appalachia as congruent with the complexity of queer identity. Together, they surface the experience of living as an insider in Appalachia while simultaneously navigating community as a queer outsider. Their essays convey the struggles unique to LGBTQ+ people in Appalachia, who sometimes consider leaving the mountains to live as an accepted queer person, only to realize that their Appalachian-ness may equate to a marginalized life elsewhere.

The book is divided into four parts, with each section taking the reader down a different, but related, path to understanding the voice of queer people in Appalachia. It begins with essays about queer-affirming and queerphobic challenges, continues to commentaries explaining the queer diaspora, moves the reader to build understanding on both the trauma and resilience of being queer in Appalachia, and concludes with essays of how queer people have worked to change their experience. The essays are powerful and underscore the key point that “acceptance with wariness” is not acceptance—but rather serves only to highlight difference. The thorough explanations within this book directly relate to the human need for love and belonging; in so doing, they create a psychological awakening for readers from Appalachia, where the culture emphasizes love, acceptance, and understanding for all people.

From failed conversion therapy to the adoption of a “don’t ask, don’t tell” posture, the essays make it clear how religion can be weaponized against LGBTQ+ Appalachians. The pain is unmistakable and the air “toxic”—referenced *not* in relation to pollution, ironically, but as a result of the often-strict religious rhetoric permeating the region. Readers are called to challenge the ACQR (Appalachian Christian Queerphobic Rhetoric), which promotes soul-crushing “fear-based hate,” and advocate for themselves and others through storytelling, “naming and claiming our space”. In doing so, queer identity becomes a part of the fabric of Appalachia, which makes it more challenging to disseminate hate.

Importntly, the authors note that this practice only succeeds if LGBTQ+ *allies* become “storylisteners,” creating room for the full experiences of their peers. Given the timeframe in which the writings occurred, many stories touch on the impact of the Trump presidency. Acknowledging “Trump Country” became synonymous with increasing the rural/urban divide and feeling a loss of “safety and rights” as bathroom bills flourished, Trans individuals were denied military service, and the president remained silent on LGBTQ+ human rights issues globally. While the Trump years galvanized many LGBTQ+ community members to action, others noted it also further isolated and displaced. The effects of this tumult can be seen in increased disparity for this specific population in regard to mental health, substance use disorders, and addiction—all currently overwhelming the Appalachian region.

#### Relevance to Appalachian Health

Not enough is known about the health of the LGBTQ+ population in Appalachia. A recent report of stakeholder meetings held by the Appalachian Regional Commission in preparation for 2022–2026 strategic planning noted that community members value diversity in Appalachia. Yet the report highlighted concern that for “those Appalachians who are a part of minority or marginalized populations, their ability to find their sense of place and community is limited.”^1^ While community members expressed that the LGBTQ+ population should be included as part of Appalachian diversity, not enough is being done to ensure full inclusion.

Despite being recognized as valued within mountain communities, LGBTQ+ individuals are underrepresented in available data and research outputs, and theory about queer health is underdeveloped as it relates to Appalachia. Consider this example: despite the rise in “grandfamilies”, especially in relation to the opioid crisis in Appalachia, a recent “State of Grandfamilies” report fails to mention—let alone address—issues relating to either potential LGBTQ+ children or grandparents.^2^ The absence of recognizing the LGBTQ+ population, while not unique to Appalachia, reinforces the lack of inclusion in working to address the region’s healthcare challenges. Furthermore, although the American Association of Colleges of Nursing (AACN) recommends that all pre- and post-licensure programs in nursing address diversity and culture as part of competent care,^3^ this does not include LGBTQ+ care as recommended by the Human Rights Campaign (HRC) in their annual Healthcare Equality Index.^4^

Yet training that is sensitive to the needs of LGBTQ+ patients can be effective in Appalachia, as it has been elsewhere. A recent study of an LGBTQ+ patient-centred care module demonstrated an increase in knowledge among care providers based on mean scores pre- and post-test and significant results in relation to attitudes and self-efficacy.^5^ The study of primary care APRNs, including midwives in Kentucky, used an online environment to facilitate learning. Additionally, although no difference was found between rural and urban providers in attitude (and knowledge scores improved overall for all), self-efficacy or confidence were lower in rural providers (but did not reach statistical significance). While the lack of significance in knowledge was thought to be related to methodology, more research is needed to confirm the author’s findings in other provider types, as well as outcomes related to LGBTQ+ health.

Another study, which evaluated pre-licensure nursing students in their awareness, skills, and attitudes concerning older LGBTQ+ adults had an educational goal of understanding that “the family is who they say they are”.^6^ The project revealed that through using case studies, learners gained knowledge and self-efficacy in providing care for LBGTQ+ populations. Although this was a Canadian study, the authors were thorough in their review of the literature, including U.S. background information and studies. Additionally, it was noted that like the U.S., less is also known about LGBTQ+ health in Canadian populations; these groups have unique health needs and perceived barriers to accessing care; and, once care is accessed, they often receive suboptimal care. Finally, as with U.S. rural populations, LGBTQ+ individuals in Canada may experience more social isolation, despite having great connectedness to their families.

Among minoritized populations, poverty—not the population—is more often the risk factor or social determinant of importance. Bias-inducing fear (real or imagined) can lead to potential gaps in care. Health disparities in LGBTQ+ populations that arise from relational and systemic bias include substance use disorder, mental health concerns, nicotine dependency, and increased risk of personal violence. Furthermore, sexual-minority adolescents are at an increased risk for depression, suicide, substance use disorder, violence, and poor academic outcomes—and states in Appalachia have large proportions of queer youth. For example, West Virginia now has the highest per capita number of teens identifying as transgender.^7^ Stories of queer Appalachian youth become more compelling when the rurality of Appalachia compounds the health risks facing LGBTQ+ teens.

These studies further contextualise the challenges of exclusion that face queer individuals—despite considerable progress towards DEI—and ways healthcare professionals might overcome them. It is clear from public health research that pernicious challenges for LGBTQ+ folk—and difficulties in articulating those challenges—persist. The same is true for Appalachians more generally. Audra Slocum, a WVU English Professor, notes “an Appalachian ‘othering’ is still taking place, the myth of an Appalachian exceptionalism that says it’s somehow different from the rest of the country. That myth, when laminated onto the language . . . means people, Appalachians included, will look for differences and maintain certain elements to sort people according to those differences.”^8^ Against this backdrop, it is critical that those in health fields work to ensure LGBTQ+ individuals in Appalachia are not further marginalized.

Improving health across Appalachia means knowing our people as they live and thrive in communities. While the essays within *Storytelling in Queer Appalachia* are presented as an exemplar of the lived experience and may seem remote from health intervention, the implications for healthcare practice are clear. Without change in healthcare education—and action towards better understanding and conveying the needs of this population—progress cannot be made. People who are queer in Appalachia should be able to discuss any and all concerns with well-prepared providers. This book offers a window into the strengths, challenges, diversity, and struggles of queer people. In offering such a view, it enhances the understanding, sensitivity, and insight needed to transform Appalachian health care into a system that can better serve *all* Appalachians, including our LGBTQ+ population.
